# Genome sequences of human cytomegalovirus strain TB40/E variants propagated in fibroblasts and epithelial cells

**DOI:** 10.1186/s12985-021-01583-3

**Published:** 2021-06-03

**Authors:** Ahmed Al Qaffas, Salvatore Camiolo, Mai Vo, Alexis Aguiar, Amine Ourahmane, Myrna Sorono, Andrew J. Davison, Michael A. McVoy, Laura Hertel

**Affiliations:** 1 Department of Pediatrics, Virginia Commonwealth University, Richmond, VA 23298 USA; 2 MRC-University of Glasgow Centre for Virus Research, Glasgow, G61 1QH UK; 3 Department of Pediatrics, School of Medicine, University of California San Francisco, Oakland, CA 94609 USA; 4 Department of Microbiology and Immunology, Virginia Commonwealth University, Richmond, VA 23298 USA

**Keywords:** Human cytomegalovirus, Epithelial cell adaptation, PacBio, Whole genome sequence, TB40/E, UL122

## Abstract

The advent of whole genome sequencing has revealed that common laboratory strains of human cytomegalovirus (HCMV) have major genetic deficiencies resulting from serial passage in fibroblasts. In particular, tropism for epithelial and endothelial cells is lost due to mutations disrupting genes *UL128*, *UL130*, or *UL131A,* which encode subunits of a virion-associated pentameric complex (PC) important for viral entry into these cells but not for entry into fibroblasts. The endothelial cell-adapted strain TB40/E has a relatively intact genome and has emerged as a laboratory strain that closely resembles wild-type virus. However, several heterogeneous TB40/E stocks and cloned variants exist that display a range of sequence and tropism properties. Here, we report the use of PacBio sequencing to elucidate the genetic changes that occurred, both at the consensus level and within subpopulations, upon passaging a TB40/E stock on ARPE-19 epithelial cells. The long-read data also facilitated examination of the linkage between mutations. Consistent with inefficient ARPE-19 cell entry, at least 83% of viral genomes present before adaptation contained changes impacting PC subunits. In contrast, and consistent with the importance of the PC for entry into endothelial and epithelial cells, genomes after adaptation lacked these or additional mutations impacting PC subunits. The sequence data also revealed six single noncoding substitutions in the inverted repeat regions, single nonsynonymous substitutions in genes *UL26*, *UL69*, *US28*, and *UL122,* and a frameshift truncating gene *UL141*. Among the changes affecting protein-coding regions, only the one in *UL122* was strongly selected. This change, resulting in a D390H substitution in the encoded protein IE2, has been previously implicated in rendering another viral protein, UL84, essential for viral replication in fibroblasts. This finding suggests that IE2, and perhaps its interactions with UL84, have important functions unique to HCMV replication in epithelial cells.

Passage of human cytomegalovirus (HCMV; species *Human betaherpesvirus 5*) in cell culture results in mutations ranging from small substitutions, insertions, or deletions to large-scale deletions, duplications, and rearrangements. Some mutations appear to be stochastic, whereas others consistently disrupt specific genes and confer growth advantages in certain cell types or growth conditions [1–3]. Of particular importance are mutations that invariably arise during passage in fibroblasts and alter one or more of three contiguous genes, *UL128*, *UL130,* and *UL131A* [1]. These mutations prevent assembly of a pentameric complex (PC) consisting of the UL128, UL130, and UL131A proteins complexed with glycoproteins H and L on the virion surface that is important for entry into epithelial and endothelial cells but dispensable for entry into fibroblasts [4–8].

Many commonly used laboratory strains have large deletions as well as various additional mutations that disrupt PC expression, thereby rendering the virus non-epitheliotropic and non-endotheliotropic [9]. Consequently, research has shifted toward the use of genetically more authentic strains such as the endothelial cell-adapted strain TB40/E [10]. However, the designation “TB40/E” has been applied indiscriminately to heterogeneous stocks propagated from the original TB40/E stock [10], as well as to a variety of viruses derived from bacterial artificial chromosome (BAC) clones generated from such stocks [11, 12]. The sequences and tropisms of these viruses can differ significantly from each other and from the original TB40/E strain. For example, clone Lisa, a virus that was plaque-purified from a TB40/E stock [13], has a 1 bp insertion in *UL128* causing a frameshift after codon 69 [13], whereas the widely utilized BAC clone TB40-BAC4 carries a single nucleotide substitution in the intron between *UL128* exons 2 and 3 that reduces splicing efficiency, lowers levels of the encoded protein, and reduces infection efficiency in epithelial cells [3]. In contrast, a more recently constructed BAC clone, TB40-KL7-SE, has no obvious mutations impacting PC expression and is both endotheliotropic and epitheliotropic [12].

To begin addressing the diversity of TB40/E stocks and the impact of propagation using different cell types, a TB40/E stock amplified twice in primary human foreskin fibroblasts (HFF; TB40/EF, stock 31,519) and entering retinal pigmented epithelial cells (ARPE-19; ATCC® CRL-2302) with poor efficiency [14] was passaged five times in ARPE-19 cells, generating a stock (TB40/EE, stock SE2) capable of infecting HFF and ARPE-19 cells with similar efficiencies [14]. Despite efficient entry, the amounts of cell-free virus generated by ARPE-19 cells infected with TB40/EE were consistently 100- to 1,000-fold lower than those produced by HFF cells, revealing the existence of epithelial cell-specific post-entry restrictions. TB40/EE also exhibited an increased propensity to form multinucleated syncytia in ARPE-19 cell populations, suggesting an enhancement in the ability to induce cell–cell fusion during infection [14].

In the current study, we used long-read PacBio sequencing to examine in detail the genetic changes potentially associated with adaptation to epithelial cells. DNA was isolated from TB40/EF and TB40/EE cell-free virions as described previously [15]. HiFi SMRTbell library construction and sequencing were performed at the Genomics Core at Virginia Commonwealth University as described previously [16]. The data were processed using tools from the PacBio SMRT-Link command-line package (https://www.pacb.com/support/software-downloads/) with default settings. Two-modal polymerase reads for TB40/EF (25,124) or TB40/EE (8,364) were indexed using pbindex, XML files of the subread counts were produced using dataset, and 16,920 HiFi reads were generated for TB40/EF and 6,985 HiFi reads for TB40/EE using CCS. The approximately three-fold difference in TB40/EF compared to TB40/EE reads is consistent with a three-fold difference in DNA concentration, which in turn reflects reduced levels of cell-free virus released from TB40/EE-infected ARPE-19 cells [14]. Final HCMV genome assemblies were made by reference-guided de novo assembly using LoReTTA v0.1 (https:/bioinformatics.cvr.ac.uk/software/; [16] with default settings and the sequence of strain TB40/E clone Lisa (13; GenBank accession no. KF297339.1) as the reference. HiFi reads were then mapped to the respective final assemblies using minimap v2.17-r941 [17], and the read alignments were visualized using the Integrative Genomics Viewer [18]. The consensus genome sequences were deposited in GenBank under accession numbers MW439038 (TB40/EF) and MW439039 (TB40/EE), and had median coverage depths of 202 and 43 reads/nucleotide, respectively. Differences between these sequences were identified, and these and other major heterogeneities noted during examination of the read alignments were quantified by counting their occurrence in the reads.

The HCMV genome (236 kbp) consists of unique long (U_L_) and unique short (U_S_) regions, each of which is flanked by inverted repeats in the arrangement *ab*-U_L_-*bʹaʹcʹ*-U_S_-*ca* (the primes denote the inverted repeats of *a*, *b*, and *c*). Comparison of the TB40/EF and TB40/EE consensus genome sequences identified 12 single nucleotide substitutions and one single nucleotide deletion within noncoding regions in the *a*, *b*, or *c* inverted repeats (Table 1). As seven of these were replicated in the inverted repeats (one each in *a/aʹ/a*, *b/bʹ*, and *c/cʹ*), these loci represent only six unique differences. Although these mutations are in noncoding regions, the significant levels of enrichment suggest that they may provide a selective advantage in ARPE-19 cells. However, the inverted repeats have been reported to be particularly prone to mutation during passage of HCMV, with changes being generally replicated in all copies presumably as the result of recombination [1].

**Table 1:** Nucleotide differences between TB40/EF and TB40/EE within non-coding regions

Region	Change*	Prevalences (%)^†^	
		TB40/EF	TB40/EE
*a/aʹ/a*	A639G/T194812C/A236421G^**‡**^	16/10/29	76/62/94
	A656C/T194795G/A236438C^**‡**^	17/10/33	94/87/100
*aʹ*	1 bp deletion at C194972	0	100
*b/bʹ*	A719C/T194732G^**‡**^	6/6	84/80
	G1174T/C194277A^**‡**^	12/6	86/85
*cʹ/c*	C195520G/G235714C^**‡**^	0/0	52/67

^*^Coordinates refer to TB40/EF; changes in TB40/EE are reported in comparison with TB40/EF

^†^Percentage of reads containing the variant

^**‡**^Mutations replicated within the *a/aʹ/a, b/bʹ*, or *cʹ/c* repeats

Comparison of the TB40/EF and TB40/EE consensus genome sequences and examination of the read alignments also identified changes in coding regions (Table 2). Given the low efficiency of epithelial cell entry observed for TB40/EF, mutations disrupting *UL128*, *UL130,* or *UL131A* (encoding the PC subunit proteins UL128, UL130, and UL131A, respectively) were anticipated. Indeed, targeted sequencing of TB40/EF had previously identified a suppressor substitution converting the *UL128* stop codon (TGA) to TTA (encoding leucine), thereby extending UL128 by 19 residues [14]. Although the consensus genome sequence of TB40/EF did not reflect this mutation, examination of read frequencies revealed the existence of a subpopulation in which 30% of TB40/EF genomes contain this suppressor mutation. Further examination identified two additional subpopulations: one containing a 2 bp deletion causing a frameshift in *UL128* and resulting in truncation of UL128 (44% of genomes), and one containing a single nucleotide substitution in *UL130* resulting in a C207S substitution in UL130 (9% of genomes) (Table 2). There was no evidence for subpopulations with mutations in *UL131A.* The long length of PacBio reads connected not only the two loci in *UL128*, which are separated by107 bp, but also the *UL130* locus 814 bp beyond; among the connecting reads, those containing one mutation did not contain the others. Thus, consistent with the low epithelial cell entry efficiency of the TB40/EF stock [14], these findings suggest that cumulatively 83% of TB40/EF genomes contain mutations potentially impacting PC assembly or function. In contrast, and consistent with efficient epithelial cell entry [14], mutations impacting PC subunits were absent from TB40/EE.

**Table 2:** Nucleotide differences between TB40/EF and TB40/EE within coding regions

Gene	Protein	Position^*^	Sequence	Consequence	Prevalence (%)^†^	
					TB40/EF	TB40/EE
*UL26*	UL26	32,663^§^	C	E98	47	0
			T	K98	53	100
*UL69*	UL69	100,074	G	H492	66	48
			C	Q492	44	52
*UL122*	IE2	171,008	C	D390	95	13
			G	H390	5	87
*UL128*	UL128	175,937^§^	A	changes stop codon to Leu	30	0
			C	wild-type	70	100
		176,044	TC	deletion introduces a frameshift after codon 135	56	0
				wild-type	44	100
*UL130*	UL130	176,753^§^	G	S207, disrupts disulfide bond with C176	9	0
			C	C207, forms disulfide bond with C176	91	100
*UL141*	UL141	184,933		wild-type	56	31
			TA	insertion introduces a frameshift after codon 63	44	69
*US28*	US28	227,251^§^	T	C320	12	0
			G	W320	88	100

^*^Coordinates refer to TB40/EF

^†^Percentage of reads containing the variant

^§^The consensus sequences for TB40/EF and TB40/EE are identical at this locus

Although it has not been demonstrated that the UL128 suppressor substitution disrupts the function of the PC, indirect evidence indicates that the UL130 C207S substitution is likely to have a negative effect. The crystal structure of the PC indicates the presence of a disulfide bond between C207 and C172 [19], and the converse mutation, C172W, has been reported to occur during serial fibroblast passage of HCMV strain IgKG-H2 in conjunction with loss of epithelial cell tropism [16]. Moreover, in HCMV strain Towne, a frameshift in *UL130* after codon 203 replaces 11 C-terminal residues (including C207) with 26 novel residues, resulting in rapid degradation of the mutant protein and loss of endothelial cell tropism [20]. These findings suggest that the C172-C207 disulfide bond is critical for the function or stability of UL130, and for its essential role in PC formation and epithelial cell entry. Curiously, the three mutations impacting PC subunit genes in TB40/EF are different from the mutations in clone Lisa or TB40-BAC4, and were not detected in reads from TB40/EE. Thus, within the available TB40/E lineages, at least five distinct mutations targeting PC subunit genes have been identified thus far.

Five other changes impacting coding regions were also identified (Table 2). These included single nonsynonymous substitutions in genes *UL26* (resulting in E98K in UL26), *UL69* (H492Q in UL69), *UL122* (D390H in IE2), and *US28* (C320W in US28), and a two nucleotide insertion in gene *UL141* introducing a frameshift truncating UL141. Examination of read frequencies revealed that most of these changes were enriched to a marginal or modest level: *UL26* (from 53 to 100%), *UL69* (from 44 to 52%), *UL141* (from 44 to 69%) and *US28* (from 88 to 100%). Thus, although these changes may be associated with improved replication in ARPE-19 cells, they may have been the consequence of stochastic effects. Moreover, both variants of *UL69* and *UL141* have been reported previously in consensus sequences of strain TB40/E, namely a partial TB40/E sequence (GenBank accession number AY446866.1), clone Lisa (KF297339.1), and the BAC clones TB40-BAC4 and TB40-KL7-SE (EF999921.1 and MF871618.1, respectively) [9, 11–13]. In *UL69*, clone Lisa and TB40-BAC4 encode Q492, whereas TB40-KL7-SE encodes H492. In *UL141*, the frameshift is absent from clone Lisa but present in the partial TB40/E sequence, TB40-BAC4, and TB40-KL7-SE. Thus, it appears that parental TB40/E stocks contained two variants of both genes, with capture of one or the other allele in the genomes of clone Lisa, TB40-BAC4, and TB40-KL7-SE resulting from cloning. In contrast, the prevalence of the D390H substitution in IE2 increased markedly from 5 to 87% (Table 2), suggesting strong selective pressure favoring this allele during ARPE-19 adaptation. The D390 allele is unique to strain TB40/E and is present in all currently reported TB40/E-derived sequences, whereas the H390 allele is conserved among all other HCMV strains for which sequences are publicly available. This observation is all the more interesting given that gene *UL84* has been identified as being essential for replication in vitro in fibroblasts in the presence of the IE2 H390 allele, but non-essential in the presence of the D390 allele [21].

In summary, whole genome sequencing identified variants impacting IE2 and PC subunits UL128 and UL130 as being potentially selected during adaptation of HCMV strain TB40/E for growth in epithelial cells. Enrichment of viral genomes lacking disruptive mutations in *UL128* and *UL130* is consistent with the detected improvement in efficiency of epithelial cell entry [14], and, as the PC has been associated with increased cell–cell fusion [22–25], may also explain the reported increase in syncytium formation [14]. It is not known how the D390H polymorphism in IE2 determines the requirement for UL84 during fibroblast replication, or whether this phenomenon also extends to epithelial cells, but selection of genomes encoding the H390 allele suggests that IE2 and, perhaps, its interplay with UL84 provide important functions that are unique to HCMV replication in epithelial cells. Construction and phenotypic characterization of viral mutants containing these genetic changes in isolation are in progress to further elucidate the role of UL84 in the context of IE2 H390 or IE2 D390.

## Data Availability

Genome sequences are available from GenBank under accession numbers MW439038 (TB40/EF) and MW439039 (TB40/EE).

## Abbreviations

HCMV: Human cytomegalovirus
PC: Pentameric complex
BAC: Bacterial artificial chromosome
HFF: Human foreskin fibroblast
U_L_: Unique long
U_S_: Unique short

## References

[1] Dargan DJ, Douglas E, Cunningham C, Jamieson F, Stanton RJ, Baluchova K, McSharry BP, Tomasec P, Emery VC, Percivalle E (2010). Sequential mutations associated with adaptation of human cytomegalovirus to growth in cell culture. J Gen Virol.

[2] Murrell I, Wilkie GS, Davison AJ, Statkute E, Fielding CA, Tomasec P, Wilkinson GW, Stanton RJ (2016). Genetic stability of bacterial artificial chromosome-derived human cytomegalovirus during culture in vitro. J Virol.

[3] Murrell I, Tomasec P, Wilkie GS, Dargan DJ, Davison AJ, Stanton RJ (2013). Impact of sequence variation in the UL128 locus on production of human cytomegalovirus in fibroblast and epithelial cells. J Virol.

[4] Hahn G, Revello MG, Patrone M, Percivalle E, Campanini G, Sarasini A, Wagner M, Gallina A, Milanesi G, Koszinowski U (2004). Human cytomegalovirus UL131-128 genes are indispensable for virus growth in endothelial cells and virus transfer to leukocytes. J Virol.

[5] Wang D, Shenk T (2005). Human cytomegalovirus virion protein complex required for epithelial and endothelial cell tropism. Proc Natl Acad Sci U S A.

[6] Adler B, Scrivano L, Ruzcics Z, Rupp B, Sinzger C, Koszinowski U (2006). Role of human cytomegalovirus UL131A in cell type-specific virus entry and release. J Gen Virol.

[7] Ryckman BJ, Rainish BL, Chase MC, Borton JA, Nelson JA, Jarvis MA, Johnson DC (2008). Characterization of the human cytomegalovirus gH/gL/UL128-131 complex that mediates entry into epithelial and endothelial cells. J Virol.

[8] Freed DC, Tang Q, Tang A, Li F, He X, Huang Z, Meng W, Xia L, Finnefrock AC, Durr E (2013). Pentameric complex of viral glycoprotein H is the primary target for potent neutralization by a human cytomegalovirus vaccine. Proc Natl Acad Sci USA.

[9] Dolan A, Cunningham C, Hector RD, Hassan-Walker AF, Lee L, Addison C, Dargan DJ, McGeoch DJ, Gatherer D, Emery VC (2004). Genetic content of wild-type human cytomegalovirus. J Gen Virol.

[10] Sinzger C, Schmidt K, Knapp J, Kahl M, Beck R, Waldman J, Hebart H, Einsele H, Jahn G (1999). Modification of human cytomegalovirus tropism through propagation in vitro is associated with changes in the viral genome. J Gen Virol.

[11] Sinzger C, Hahn G, Digel M, Katona R, Sampaio KL, Messerle M, Hengel H, Koszinowski U, Brune W, Adler B (2008). Cloning and sequencing of a highly productive, endotheliotropic virus strain derived from human cytomegalovirus TB40/E. J Gen Virol.

[12] Sampaio KL, Weyell A, Subramanian N, Wu Z, Sinzger C (2017). A TB40/E-derived human cytomegalovirus genome with an intact US-gene region and a self-excisable BAC cassette for immunological research. Biotechniques.

[13] Tomasec P, Wang EC, Davison AJ, Vojtesek B, Armstrong M, Griffin C, McSharry BP, Morris RJ, Llewellyn-Lacey S, Rickards C (2005). Downregulation of natural killer cell-activating ligand CD155 by human cytomegalovirus UL141. Nat Immunol.

[14] Vo M, Aguiar A, McVoy MA, Hertel L. Cytomegalovirus Strain TB40/E Restrictions and Adaptations to Growth in ARPE-19 Epithelial Cells. *Microorganisms* 2020, 8. 10.3390/microorganisms8040615 PMC7232150 32344555

[15] Ourahmane A, Cui X, He L, Catron M, Dittmer DP, Al Qaffasaa A, Schleiss MR, Hertel L, McVoy MA. Inclusion of Antibodies to Cell Culture Media Preserves the Integrity of Genes Encoding RL13 and the Pentameric Complex Components During Fibroblast Passage of Human Cytomegalovirus. *Viruses* 2019, 11. 10.3390/v11030221 PMC6466449 30841507

[16] Qaffas AA, Nichols J, Davison AJ, Ourahmane A, Hertel L, McVoy MA, Camiolo S. LoReTTA, a user-friendly tool for assembling viral genomes from PacBio sequence data. *Virus Evolution* 2021. 10.1093/ve/veab042 PMC8111061 33996146

[17] Li H (2018). Minimap2: pairwise alignment for nucleotide sequences. Bioinformatics.

[18] Robinson JT, Thorvaldsdottir H, Winckler W, Guttman M, Lander ES, Getz G, Mesirov JP (2011). Integrative genomics viewer. Nat Biotechnol.

[19] Chandramouli S, Malito E, Nguyen T, Luisi K, Donnarumma D, Xing Y, Norais N, Yu D, Carfi A. Structural basis for potent antibody-mediated neutralization of human cytomegalovirus. *Sci Immunol* 2017, 2. 10.1126/sciimmunol.aan1457 28783665

[20] Patrone M, Secchi M, Fiorina L, Ierardi M, Milanesi G, Gallina A (2005). Human cytomegalovirus UL130 protein promotes endothelial cell infection through a producer cell modification of the virion. J Virol.

[21] Spector DJ (2015). UL84-independent replication of human cytomegalovirus strains conferred by a single codon change in UL122. Virology.

[22] Cui X, Freed DC, Wang D, Qiu P, Li F, Fu TM, Kauvar LM, McVoy MA: Impact of Antibodies and Strain Polymorphisms on Cytomegalovirus Entry and Spread in Fibroblasts and Epithelial Cells. *J Virol* 2017, 91. 10.1128/JVI.01650-16 PMC5469265 28381568

[23] Chiuppesi F, Wussow F, Johnson E, Bian C, Zhuo M, Rajakumar A, Barry PA, Britt WJ, Chakraborty R, Diamond DJ (2015). Vaccine-derived neutralizing antibodies to the human cytomegalovirus gH/gL pentamer potently block primary cytotrophoblast infection. J Virol.

[24] Ciferri C, Chandramouli S, Donnarumma D, Nikitin PA, Cianfrocco MA, Gerrein R, Feire AL, Barnett SW, Lilja AE, Rappuoli R (2015). Structural and biochemical studies of HCMV gH/gL/gO and Pentamer reveal mutually exclusive cell entry complexes. Proc Natl Acad Sci USA.

[25] Gerna G, Percivalle E, Perez L, Lanzavecchia A, Lilleri D (2016). Monoclonal antibodies to different components of the human cytomegalovirus (HCMV) pentamer gH/gL/pUL128L and trimer gH/gL/gO as well as antibodies elicited during primary HCMV infection prevent epithelial cell syncytium formation. J Virol.

